# EGFR and EGFRvIII undergo stress- and EGFR kinase inhibitor-induced mitochondrial translocalization: A potential mechanism of EGFR-driven antagonism of apoptosis

**DOI:** 10.1186/1476-4598-10-26

**Published:** 2011-03-09

**Authors:** Xinyu Cao, Hu Zhu, Francis Ali-Osman, Hui-Wen Lo

**Affiliations:** 1 Division of Surgical Sciences, Department of Surgery, Duke University School of Medicine, 433A MSRB I, 103 Research Drive, Durham, NC 27710, USA; 2 Duke Comprehensive Cancer Center, Durham, NC 27710, USA; 3 Preston Robert Tisch Brain Tumor Center at Duke University, Durham, NC 27710, USA; 4 Department of Pharmacology, Box 7365, University of North Carolina-Chapel Hill, Chapel Hill, NC 27599, USA

## Abstract

**Background:**

Epidermal growth factor receptor (EGFR) plays an essential role in normal development, tumorigenesis and malignant biology of human cancers, and is known to undergo intracellular trafficking to subcellular organelles. Although several studies have shown that EGFR translocates into the mitochondria in cancer cells, it remains unclear whether mitochondrially localized EGFR has an impact on the cells and whether EGFRvIII, a constitutively activated variant of EGFR, undergoes mitochondrial transport similar to EGFR.

**Results:**

We report that both receptors translocate into the mitochondria of human glioblastoma and breast cancer cells, following treatments with the apoptosis inducers, staurosporine and anisomycin, and with an EGFR kinase inhibitor. Using mutant EGFR/EGFRvIII receptors engineered to undergo enriched intracellular trafficking into the mitochondria, we showed that glioblastoma cells expressing the mitochondrially enriched EGFRvIII were more resistant to staurosporine- and anisomycin-induced growth suppression and apoptosis and were highly resistant to EGFR kinase inhibitor-mediated growth inhibition.

**Conclusions:**

These findings indicate that apoptosis inducers and EGFR-targeted inhibitors enhance mitochondrial translocalization of both EGFR and EGFRvIII and that mitochondrial accumulation of these receptors contributes to tumor drug resistance. The findings also provide evidence for a potential link between the mitochondrial EGFR pathway and apoptosis.

## Background

EGFR is an important mediator of normal cell growth and differentiation [1,2]. In cancer cells, EGFR is frequently over-expressed and is associated with tumor proliferation, progression and drug resistance [3-5]. EGFRvIII, a constitutively activated EGFR variant, is a product of rearrangement with an in-frame deletion of 801 bp of the coding sequence of the EGFR extracellular domain that results in a deletion of residues 6 through 273 and a glycine insertion as residue 6 [6-9]. EGFR/EGFRvIII gene amplification is frequent in glioblastoma multiforme (GBM), the most common and deadliest brain cancer in adults [9,10]. Consequently, both EGFR and EGFRvIII are being targeted for cancer therapy [3,11,12].

The anticancer efficacy of anti-EGFR small molecule inhibitors and monoclonal antibodies has been evaluated in clinical trials both as single agent and in combination with other chemotherapeutic agents, but to date, have shown only modest effects [13-18]. Much effort is thus being directed at understanding the mechanisms that underlie tumor resistance to anti-EGFR therapy. For example, we have recently shown that nuclear EGFR interacts with STAT3 and that the interaction contributes to tumor resistance to the anti-EGFR agent, Iressa, in human GBM [12] and breast cancer cells [19]. In addition, it has been recently reported that EGFR and EGFRvIII interacts with apoptotic protein PUMA and inhibits PUMA's apoptotic function [20]. PTEN loss has also been implicated in resistance to EGFR inhibition, although, other studies did not find such a linkage [17,18,21,22]. In lung cancer, gain-of-function EGFR mutations have been shown to be predictive of sensitivity to EGFR-targeted treatments, however, in other tumor types, these mutations are either absent or are very rare. The biology underlying tumor resistance to EGFR-targeted therapy is thus complex and remains not well understood.

An area of EGFR-associated biology in human cancers that is receiving increasing attention is the ability of EGFR to escape lysosome-mediated degradation and recycling to the plasma membranes and, subsequently, to undergo intracellular trafficking to subcellular organelles, such as, nuclei [4,19,23-25] and mitochondria [26,27]. Nuclear EGFR and mitochondrial EGFR are expressed as the full-length proteins, in contrast to HER4/ErbB4 which enters nuclei and mitochondria as its C-terminal fragment. While the cellular functions and role of nuclear EGFR are becoming clearer, those of mitochondrial EGFR are still largely unknown. Also unknown is whether EGFRvIII undergoes mitochondrial translocalization. Nevertheless, it has been shown that EGF stimulation enhances EGFR mitochondrial localization in MDA-MB-231 breast cancer cells [26] and that mitochondrial EGFR interacts with cytochrome c oxidase subunit II (CoxII) in an EGFR Y845-dependent manner [27]. EGFR Y845 is a specific phosphorylation residue targeted by c-Src and interestingly, c-Src appears to also undergo mitochondrial import with kinetics similar to that of EGFR [27]. In the mitochondria, both EGFR and c-Src can phosphorylate Cox II, albeit the consequence of this phosphorylation remains unclear [27].

Given the pivotal role that mitochondria plays in intrinsic apoptosis, we investigated, in this study, the effects of apoptosis-inducing agents on mitochondrial translocalization of both EGFR and EGFRvIII. We also conducted a series of experiments to address the impact of the mitochondrial accumulation of EGFR and EGFRvIII on the apoptotic response of cancer cells treated with apoptosis-inducing agents and an EGFR kinase inhibitor, Iressa. Our findings demonstrate that both EGFR and EGFRvIII undergo mitochondrial translocalization when cancer cells encounter apoptotic stimuli. Using cells that stably express EGFRvIII and mitochondrially enriched EGFRvIII mutant, we found that mitochondrial accumulation of EGFRvIII rendered the cells highly resistant to apoptosis induced by these agents. These results implicate mitochondrial EGFR/EGFRvIII in the modulation of mitochondria-mediated apoptosis.

## Results

### EGFR mitochondrial translocalization is enhanced by apoptotic inducers and an EGFR kinase inhibitor

**Figure 1:**
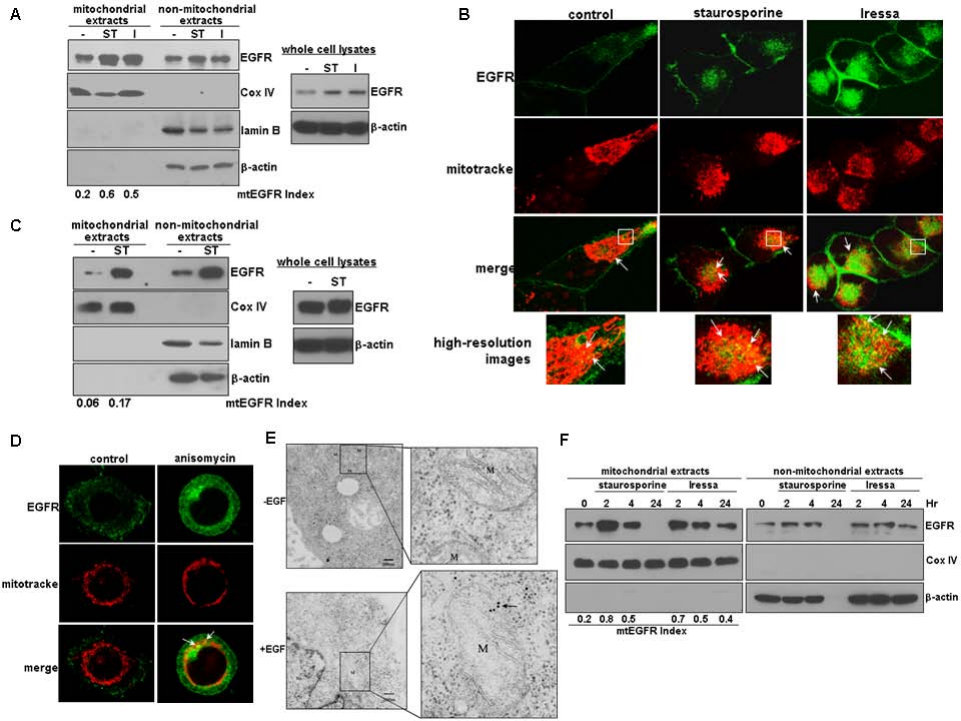
**EGFR undergoes enhanced mitochondrial translocalization after exposures to apoptosis inducers and the EGFR kinase inhibitor, Iressa**. ***A***, An apoptotic inducer and an EGFR kinase inhibitor enhance EGFR mitochondrial transport. Human T98G GBM cells with high endogenous levels of EGFR were treated with and without 1 uM staurosporine (ST) and 12.5 uM Iressa (I) for 15 min, fractionated into mitochondrial and non-mitochondrial fractions, and analyzed by western blotting. The extent of EGFR mitochondrial translocalization was indicated by mtEGFR index. Right panel: western blots with whole cell lysates. ***B***, Immunofluorescence staining/confocal microscopy showing staurosporine- and Iressa-induced EGFR mitochondrial translocalization. T98G cells were similarly treated as in Panel A, fixed and subjected to immunofluorescence staining/confocal microscopy to mark mitochondrial EGFR. Mitochondrial EGFR (arrows) is shown as the yellow signals merged from the green fluorescence (EGFR) and the red fluorescence (mitotracker, a mitochondria-specific dye). Bottom panels: high-resolution images. ***C***, Endogenous EGFR in breast cancer MDA-MB-468 cells undergoes staurosporine-induced mitochondrial transport. The cells were treated with and without staurosporine (ST) for 15 min, fractionated and subjected to western blotting. Right panel shows western blots with whole cell lysates. ***D***, Anisomycin stimulates EGFR mitochondrial translocalization in MDA-MB-468 cells. The cells were treated with and without 100 ng/ml anisomycin for 15 min and subjected to immunofluorescence staining/confocal microscopy, as described earlier in Panel B. Arrows: representative EGFR signals in the mitochondria. ***E***, EM shows mitochondrial presence of EGFR in EGF-treated tumor cells. Arrows point to EGFR signals in the mitochondria. Right panels show high-resolution images. Mitochondrion is labeled as M. Nucleus is labeled as Nu. ***F***, Staurosporine and Iressa induced EGFR mitochondrial transport in a time-dependent fashion peaking at 2 hrs after treatments. The majority of T98G cells did not survive 24 hr staurosporine treatment as indicated by the lack of β-actin and EGFR expression.

The impact of apoptotic stimuli on EGFR mitochondrial translocalization remains uninvestigated. Using human GBM T98G cells that express high endogenous levels of EGFR, we found that full-length EGFR undergoes increased mitochondrial translocalization after treatments with staurosporine (ST) and Iressa (I) for 15 min (Figure 1A; left panel). Cell fractionation was effective as indicated by the lack of the cytoplasmic marker, β-actin, and the nuclear protein, lamin B, in the mitochondrial extracts, as well as, by the absence of the mitochondrial protein, Cox IV, in the non-mitochondrial extracts. As shown by the right panel of Figure 1A, both treatments have led to an increased level of total EGFR, suggesting that they may regulate EGFR expression at the post-translational level. The extent of EGFR mitochondrial translocalization was subsequently computed and expressed as an mtEGFR index. Notably, staurosporine and Iressa increased the mtEGFR index by approximately 2.5- to 4-fold (Figure 1A). This was further confirmed using immunofluorescence staining and confocal microscopy (Figure 1B), in which mitochondrial EGFR was marked by the yellow fluorescence signals (arrows) merged from the green fluorescence (EGFR) and red fluorescence (mitochondria-specific dye, mitotracker). The bottom panels of Figure 1B show high-resolution images in which arrows point to mitochondrial EGFR (yellow signals). The observations made in T98G cells were similarly found in cells of the human breast cancer, MDA-MB-468, that express endogenous EGFR (Figures 1C-1E), in which the two apoptosis-inducers, staurosporine and anisomycin, were used to stress the cells. As shown by the right panel of Figure 1C, staurosporine has modestly increased the level of total EGFR. Using electron microscopy (Figure 1E), we observed that EGFR was readily detected in the mitochondria of EGF-treated tumor cells in which arrows mark gold particles that label EGFR. As shown in Figure 1F, we further found that staurosporine and Iressa induced EGFR mitochondrial transport in a time-dependent fashion peaking at 2 hrs after treatments. Notably, the majority of T98G cells did not survive 24 hr staurosporine treatment as indicated by the lack of β-actin and EGFR expression. Noticeably, both the GBM and breast cancer cell lines we analyzed contained a basal level of mitochondrial EGFR without stimulation. This may be due to an autocrine effect given the ability of EGF to induce EGFR mitochondrial translocalization [26]. Together, these results indicate that apoptotic stimuli enhance EGFR mitochondrial translocalization in human cancer cells.

### EGFRvIII undergoes mitochondrial translocalization and the degree of transport is enhanced by apoptosis inducers and Iressa

**Figure 2:**
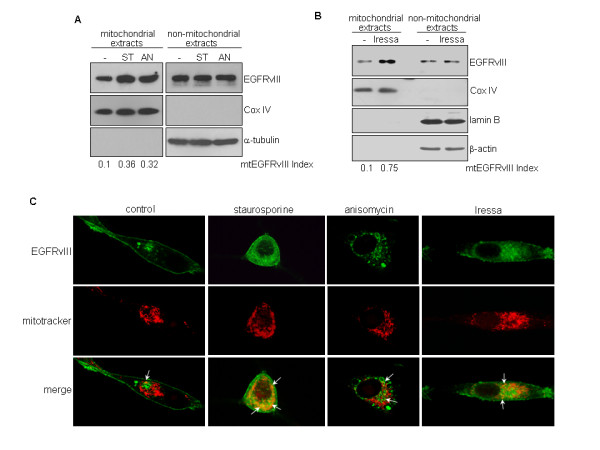
**EGFRvIII translocates into mitochondria even under unstressed condition but the transport is enhanced by apoptosis inducers and EGFR inhibition**. Since endogenous EGFRvIII expression is not maintained *in vitro *[28], we used U87MG-EGFRvIII stable transfectant cells expressing EGFRvIII [12] in these studies. ***A***, Full-length EGFRvIII is present in the mitochondria under unstressed condition and the level is enhanced by apoptotic stress and Iressa. Cancer cells treated with and without 1 uM staurosporine (ST) and 100 ng/ml anisomycin (AN) for 15 min were fractionated into mitochondrial and non-mitochondrial fractions followed by western blotting. Full-length EGFRvIII was detected in both extracts. The mtEGFRvIII indices indicate that staurosporine and anisomycin increased the levels of mitochondrial EGFRvIII by approximately 3-4 folds. ***B***, Iressa induces significant mitochondrial transport of EGFRvIII. Cancer cells were treated with and without 12.5 uM Iressa, an EGFR kinase inhibitor, for 15 min and subjected to cell fractionation and western blotting. Effectiveness of cell fractionation was indicated by the lack of the cytoplasmic marker β-actin and a nuclear protein lamin B in the mitochondrial extracts, as well as, by the absence of the mitochondrial protein Cox IV in the non-mitochondrial extracts. The mtEGFRvIII indices indicate that Iressa resulted in a marked increase (7.5-fold) of mitochondrial EGFRvIII. ***C***, Immunofluorescence staining and confocal microscopy confirmed staurosporine-, anisomycin and Iressa-induced EGFRvIII mitochondrial import. In these studies, we used an EGFR antibody that recognized the N-terminal epitope in EGFRvIII and the Myc antibody that bound to the C-terminal Myc-tag of the EGFRvIII fusion protein (data not shown) and found both antibodies to detect mitochondrial EGFRvIII. This observation and the molecular weight shown in western blotting suggest that the mitochondrially localized EGFRvIII is highly likely to be the full-length protein.

It remains uninvestigated whether EGFRvIII translocates into the mitochondria. Using cell fractionation and western blotting, we show that EGFRvIII is present in the mitochondria even under unstressed condition but that the level is enhanced by apoptotic stress (Figure 2A). The molecular weight of the mitochondrially sequestrated EGFRvIII is approximately 145 kD, similar to the full-length EGFRvIII. In these studies, we used U87MG-EGFRvIII cells that we previously established to express stably transfected EGFRvIII [12] because endogenous EGFRvIII expression is not maintained *in vitro *[28]. As indicated by the mtEGFRvIII indices, staurosporine (ST) and anisomycin (AN) increased the levels of mitochondrial EGFRvIII by approximately 3-4 fold, while Iressa resulted in a 7.5-fold increase in EGFRvIII mitochondrial transport (Figure 2B). These observations were confirmed by immunofluorescence staining and confocal microscopy (Figure 2C). In the immunofluorescence staining experiments, we used an EGFR antibody that recognized the N-terminal epitope present in both EGFR and EGFRvIII (Figure 2C) and a Myc-tag antibody that bound to the C-terminal Myc-tag of the EGFR/EGFRvIII fusion proteins (data not shown). The results showed that both antibodies labeled mitochondrial EGFRvIII. This observation combined with that of the western blotting (Figure 2A) strongly indicate that the mitochondrially localized EGFRvIII is likely to be the full-length EGFRvIII.

### Amino acid substitutions of N-terminal conserved motifs increase mitochondrial import of both EGFR and EGFRvIII

**Figure 3:**
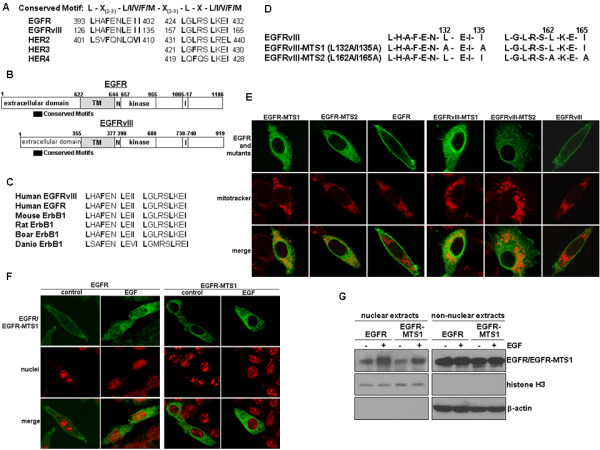
**Amino acid substitutions of the N-terminal conserved motifs enhance mitochondrial targeting of both EGFR and EGFRvIII**. ***A***, Structures of the two N-terminal conserved motifs within EGFR and EGFRvIII. The two motifs are homologous to the L/I/V/F/M-rich sequence that has been shown to involve in protein intracellular trafficking [29-31]. Notably, the first motif is present in EGFR, EGFRvIII and HER2 while the second motif is found in all members of the EGFR family of receptors. ***B***, Simplified structures of EGFR/EGFRvIII show that both sequences are present in the extracellular region of both receptors. TM, transmembrane domain. N, nuclear-localization signal. I, internalization domain. ***C***, Both motifs are conserved among EGFR proteins in different mammalian species. ***D***, EGFRvIII mutants with amino acid substitutions within the conserved motifs. To determine the role of the motifs in modulating EGFR/EGFRvIII intracellular trafficking, we performed site-directed mutagenesis to substitute leucine/isoleucine residues to alanines, thereby altering the L/I-rich property of the motifs. ***E***, EGFR-MTS and EGFRvIII-MTS mutants demonstrate enhanced degrees of mitochondrial localization. Using immunofluorescence staining/confocal microscopy, we examined subcellular location of EGFR/EGFRvIII and all four mutants in EGFR/EGFRvIII-null CHO cells. The yellow signals indicate mitochondrially localized receptors, merged products of the green fluorescence (receptors) and red fluorescence (mitochondrion labeled by mitotracker). ***F,G***, The EGFR-MTS1 mutant did not display enhanced nuclear accumulation as shown by immunofluorescence staining and confocal microscopy (Panel F) and nuclear fractionation followed by western blotting (Panel G). Transfected CHO cells were serum-starved and treated with and without EGF (100 ng/ml) for 10 min to induce EGFR nuclear import. The results showed that the EGFR MTS mutants did not have enhanced nuclear accumulation, in contrast to their ability to richly localize in the mitochondria (EGFR-MTS2 data not shown).

In this study, we targeted two N-terminal regions of EGFR and EGFRvIII that are homologous to L/I/V/F/M-rich sequences that have been shown to be involved in protein intracellular trafficking [29-31]. As shown in Figures 3A and 3B, the two N-terminal motifs are present in the extracellular region of EGFR, EGFRvIII and HER2 while the second motif is found in all members of the EGFR family of receptors. Both motifs are conserved among EGFR proteins in different mammalian species (Figure 3C). To determine the role of these motifs in modulating EGFR/EGFRvIII intracellular trafficking, we performed site-directed mutagenesis to substitute leucine/isoleucine residues with alanines and thereby, altered the L/I-rich property of the motifs. Consequently, we generated two EGFR mutants, EGFR-MTS1 (L399A/I402A) and EGFR-MTS2 (L429A/I432A) and two EGFRvIII mutants (Figure 3D), EGFRvIII-MTS1 (L132A/I35A) and EGFRvIII-MTS2 (L162A/I165A). The subcellular location of these EGFR/EGFRvIII mutants was then examined via immunofluorescence staining/confocal microscopy following transfecting the mutant constructs into EGFR/EGFRvIII-null Chinese hamster ovary (CHO) cells. The results (Figure 3E) showed that all four mutant receptors were significantly localized in the mitochondria, as indicated by the yellow signals after merging the green fluorescence (EGFR/EGFRvIII and their mutants) with the red fluorescence (mitochondrion). EGFR and EGFRvIII MTS mutant proteins were also present in the cytoplasm. Interestingly, the MTS mutants were rarely found on the plasma membranes. Given the similarity of the two N-terminal motifs to the reported nuclear-export signals [30], we determined whether the mutations within the motifs block EGFR nuclear export and consequently, facilitate EGFR nuclear accumulation. The results of immunofluorescence staining and confocal microscopy (Figure 3F) showed no increased accumulation of EGFR-MTS1 or EGFR-MTS2 (not shown) in the nucleus, despite their ability to undergo significant mitochondrial import. This was confirmed using nuclear fractionation followed by western blotting (Figure 3G), in which the effectiveness of nuclear fractionation was indicated by the absence of the cytoplasmic protein β-actin in the nucleus and the lack of the nuclear protein histone H3 in the non-nuclear extracts. Collectively, these results indicate that the EGFR/EGFRvIII MTS mutants undergo enhanced mitochondrial translocalization. These data also suggest that these mutants can be used as valuable tools for studies aiming to elucidate the actions of mitochondrial EGFR/EGFRvIII that are largely unknown at the present time.

### Tumor cells expressing the mitochondrially enriched EGFRvIII are more resistant to mitochondria-mediated apoptosis

**Figure 4:**
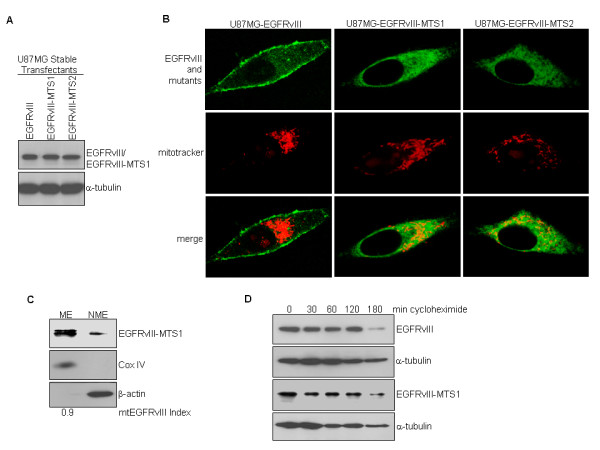
**Cancer cells expressing the mitochondrially enriched EGFRvIII mutant are more resistant to apoptosis**. ***A***, Generation of U87MG stable transfectant cell lines that express EGFRvIII-MTS1 and EGFRvIII-MTS2. Western blotting shows that the three isogenic cell lines express equivalent levels of the transgenes. ***B,C***, The EGFRvIII-MTS1 mutant is enriched in the mitochondria as shown by immunofluorescence staining and confocal microscopy (B) and cell fractionation/western blotting (C). ***D***, Stability of EGFRvIII and EGFRvIII-MTS1 proteins is similar. Protein half-life study using transcription inhibitor cycloheximide determined the two proteins to have similar degradation rates.

**Figure 5:**
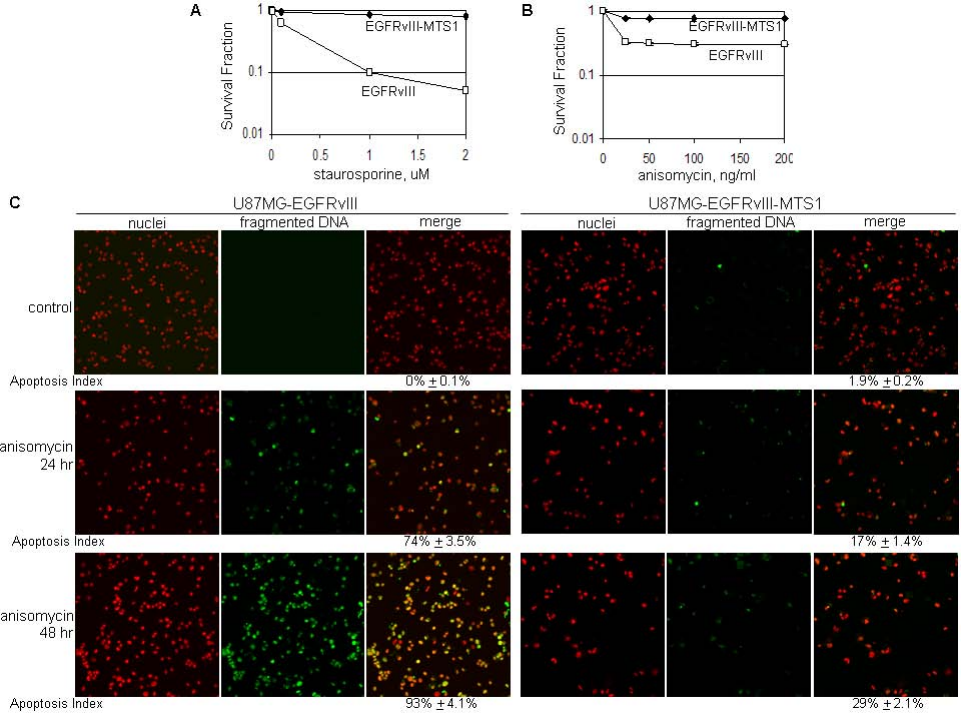
**U87MG-EGFRvIII-MTS1 cells were more resistant to apoptosis inducers than U87MG-EGFRvIII cells**. ***A,B***, U87MG-EGFRvIII-MTS1 cells survived better than U87MG-EGFRvIII cells after treatments of apoptosis inducers. The cells were exposed to staurosporine (0-2 uM; Panel A) and anisomycin (0-200 ng/ml; Panel B) for 24 and 48 hrs and the survival rates determined using the Celltiter Blue Cell Survival Assay (Promega). ***C***, U87MG-EGFRvIII-MTS1 cells are more resistant to anisomycin-induced apoptosis than U87MG-EGFRvIII cells. The extent of apoptosis was determined via the TUNEL assay, in which fragmented DNA is indicated by the yellow fluorescence signals, merged products of the red fluorescence (nuclei) and the green fluorescence (fragmented DNA).

In light of our observations that apoptotic stimuli induced EGFR/EGFRvIII translocation into the mitochondria, a focal point of intrinsic apoptosis initiation, we speculated that mitochondrial EGFR/EGFRvIII may be involved in mediating mitochondria-mediated apoptosis. Consequently, we created two U87MG stable transfectant cell lines, U87MG-EGFRvIII-MTS1 and U87MG-EGFRvIII-MTS2, to respectively express EGFRvIII-MTS1 and EGFRvIII-MTS2 mutants. As shown in Figure 4A, the expression levels of the transgenes in U87MG-EGFRvIII-MTS1 and U87MG-EGFRvIII-MTS2 stable transfectant cells were similar to that of U87MG-EGFRvIII cells. The two MTS mutants are expressed at high levels in the mitochondria as shown by the results of immunofluorescence staining/confocal microscopy (Figure 4B) and cell fractionation/western blotting (Figure 4C; only EGFR-MTS1 data are shown; ME, mitochondrial extracts; NME, non-mitochondrial extracts). Stability of EGFRvIII, EGFRvIII-MTS1 and EGFRvIII-MTS2 proteins was similar, as indicated by the protein degradation study using the transcription inhibitor, cycloheximide (Figure 4D; data for EGFRvIII-MTS2 are not shown). Given the observation that EGFRvIII-MTS1 and EGFRvIII-MTS2 behave similarly, we focused on EGFRvIII-MTS1 for determining the effects of EGFRvIII mitochondrial accumulation on the response of cancer cells to apoptosis-inducing agents. We observed that U87MG-EGFRvIII-MTS1 cells were more resistant to 48 hr treatments of staurosporine (Figure 5A) and anisomycin (Figure 5B) than U87MG-EGFRvIII cells. Consistent with these observations, anisomycin induced a significantly lower level of apoptosis in U87MG-EGFRvIII-MTS1 cells than in U87MG-EGFRvIII cells (Figure 5C). In the TUNEL assay, the extent of apoptosis was indicated by the yellow fluorescence signals, merged products of the red fluorescence (nuclei) and the green (fluorescence fragmented DNA). Together, these results suggest that EGFRvIII mitochondrial accumulation protects cancer cells from undergoing mitochondria-mediated apoptotic death.

### Translocation of EGFRvIII to mitochondria renders tumor cells resistant to EGFR inhibition

**Figure 6:**
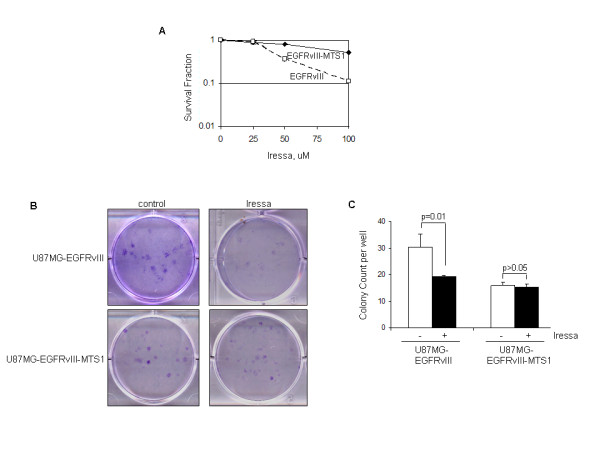
**Expression of the mitochondrially enriched EGFRvIII renders GBM cells more resistant to the EGFR inhibitor, Iressa**. ***A***, U87MG-EGFRvIII-MTS1 cells are significantly more resistant to a 48-hr Iressa treatment (0-100 uM) than U87MG-EGFRvIII cells. Survival rates were determined by the Celltiter Blue Cell Survival Assay. ***B,C***, Clonogenic growth assay confirmed the results of Panel A. The assay was performed in 6-well cell culture plates as previously described [19]. Seeded cells were treated with 1% DMSO or 12.5 μM Iressa in 1% DMSO for 24 hrs followed by medium replacement with fresh drug-free growth medium and culturing for 10-14 days. Colonies were stained with crystal violet blue solution for 1 hr, washed with water, dried and counted. Triplicate wells were used for each treatment and three independent experiments were performed to derive means and standard deviations. Student *t*-test was performed to compute p-values. Notably, U87MG-EGFRvIII-MTS1 cells are significantly more resistant to Iressa-mediated cell kill than U87MG-EGFRvIII cells. It is also noticeable that under the unstressed condition, U87MG-EGFRvIII-MTS1 cells had a reduced ability to form colonies compared to U87MG-EGFRvIII cells.

The results summarized in Figure 2 indicate that the EGFR inhibitor, Iressa, enhances EGFRvIII mitochondrial translocalization in tumor cells. We, therefore, examined whether the mitochondrial accumulation of EGFRvIII impacts cancer cell sensitivity to Iressa. The results of these studies, summarized in Figure 6A, show that U87MG-EGFRvIII-MTS1 cells with increased mitochondrial EGFRvIII are significantly more resistant to a 48-hr Iressa treatment than control U87MG-EGFRvIII cells. This is further confirmed by the results of the clonogenic cell assay (Figures. 6B and 6C), showing that control U87MG-EGFRvIII-MTS1 cells had a reduced ability to form colonies compared to untreated U87MG-EGFRvIII cells. This interesting observation may be due to the likelihood that EGFRvIII-MTS1 is mitochondrially enriched for the purpose of enhancing survival and that it is rarely localized on the cell-surface where the mitogenic proliferative signal is transmitted. These results combined with those in Figure 2 indicate that Iressa induces EGFRvIII mitochondrial translocalization and that the mitochondrial accumulation of EGFRvIII contributes to the resistance of GBM cells to EGFR inhibition.

## Discussion

We report in this study that both EGFR and EGFRvIII translocate into mitochondria upon exposure of GBM and breast cancer cells to apoptosis inducers and EGFR inhibitors and that accumulation of EGFRvIII in mitochondria renders GBM cells more resistant to the EGFR inhibitor, Iressa, and other apoptosis inducers. Two modes of EGFR signaling, cell-surface and nuclear, have been well characterized to play important roles in human cancers [3-5]. The cell-surface EGFR signaling pathway occurs through transduction of mitogenic signals from the cell-surface to PLC-γ-CaMK/PKC, Ras-Raf-MAPK, PI-3K-Akt-GSK, and JAK/STATs, and downstream to targets in the cytoplasm and the cell nucleus. The nuclear EGFR pathway involves EGFR nuclear transport and can be activated by ligand binding, radiation, cisplatin, heat- and oxidative-stress, and inhibited by exposure to vitamin D [4,32,33]. Nuclear EGFR functions as a transcription factor and tyrosine kinase, leading to increased proliferation and poor clinical outcomes in cancer [4,19,25,33-36]. Our findings in this study provide for and extend the previously described mitochondrial EGFR signaling by demonstrating the functional consequences of mitochondrial EGFR translocalization, specifically, its impact on apoptosis and drug resistance.

Our findings confirm those in previous reports [26,27,37] indicating that the full-length EGFR translocates into the mitochondria. We also demonstrate, for the first time, that EGFRvIII also translocates to mitochondria as a full-length protein. This contrasts with HER2, which has been shown to exist as both full-length (185 kD) and truncated (155 kD) proteins in the mitochondria of normal kidney and breast cells, and in SK-BR-3 breast cancer cells [38]. In contrast to EGFR and HER2, HER4 undergoes γ-secretase processing [39] and the resulting intracellular domain translocates into the mitochondria [40]. In the case of EGFR, the full-length receptor has been shown to exist in the cytoplasm as free non-membrane-bound form [41] and this is the consequence of Sec61β translocon-mediated retro-translocation that extract membrane-bound EGFR from the ER to the cytoplasm as a non-membrane-bound receptor [41]. In this form, EGFR interacts with importin β1, a nuclear import protein, crosses through nuclear pore complex into the nucleus, where it functions as a transcription factor and tyrosine kinase [35,42]. We recently showed that the non-membrane-bound cytoplasmic EGFR/EGFRvIII interacts with the pro-apoptotic protein PUMA and traps PUMA in the cytoplasm [20]. These observations are in line with the notion that full-length EGFR translocates into the mitochondria, as shown in this study and in previous other reports [26,27,37].

It remains unknown how EGFR or EGFRvIII translocate into mitochondria, particularly, since both receptors lack any of the known mitochondrial targeting signal (MTS). Interestingly, a number of cytoplasmic and nuclear proteins have been shown to undergo mitochondrial translocalization including, HER2 that lacks MTS. Similarly, p53 does not contain a MTS but translocates into the mitochondria in response to DNA damage, leading to apoptosis [43-45]. The transmembrane glycoprotein, MUC1, also lacks a MTS but undergoes proteolytic cleavage in ER, retro-translocated into cytoplasm and subsequently, its C-terminal peptide, MUC1-C localizes to the mitochondria [46]. It has been shown that MUC1-C forms intracellular complexes with chaperone HSP70/HSP90 complex [47] which enables its mitochondrial trafficking. In light of these observations, future investigations are warranted to explore whether EGFR/EGFRvIII utilizes a similar chaperone mechanism to gain entry into mitochondria.

Emerging evidence indicates that cell-surface and nuclear proteins translocate into mitochondria and that while in the mitochondria, elicit biological effects that are uniquely distinct from their cell-surface and nuclear functions. For example, cell-surface receptors (EGFR, EGFRvIII and HER2), cytoplasmic proteins (c-Src, intracellular domains of HER4 and MUC-1) and nuclear proteins (p53) can be shuttled into mitochondria and some of them have been shown to elicit unique functions [26,27,37,38,43-48]. We show in this study that mitochondrial transport of EGFR and EGFRvIII can be induced by apoptosis-inducing agents and EGFR inhibitors and that tumor cells with accumulated mitochondrial EGFRvIII are resistant to apoptosis induced by these agents. It has been previously shown that EGF stimulates EGFR mitochondrial import leading to mitochondrial interaction of EGFR with Cox II; the cellular consequence of the interaction, however, remains unclear [26,27]. The role of HER2 in the mitochondria is unknown. C-terminal region of HER4 binds to and antagonizes anti-apoptotic protein Bcl-2 and thereby promotes apoptosis of breast cancer cells [40]. Mitochondrial MUC1 has been shown to prevent cisplatin-induced apoptosis [46]. Mitochondrial p53 binds to anti-apoptotic protein Bcl-xL and induces apoptosis [43-45]. It is worth noting that a recent study by Yue et al [37] showed that EGFR mitochondrial translocation can be increased by the mTOR inhibitor, rapamycin, and decreased by the topoisomerase inhibitor, etoposide, and by 3'-methyladenine, an inhibitor of autophagy. The reported link between autophagy and mitochondrial EGFR is particularly interesting and is in line with our observation that apoptotic stress induces EGFR mitochondrial transport. Together, the results of Yue et al [37] and the current study implicate mitochondrial EGFR in apoptosis and autophagy of therapy-challenged tumor cells. These findings also call for additional efforts to elucidate the exact actions of mitochondrially localized EGFR and EGFRvIII and their impact on tumor response to anti-cancer drugs that often induce apoptosis and/or autophagy.

## Conclusions

Our findings in this study indicate that apoptosis inducers and EGFR-targeted inhibitors enhance mitochondrial translocalization of both EGFR and EGFRvIII and that mitochondrial accumulation of these receptors contributes to tumor drug resistance. The findings also provide evidence for a potential link between the mitochondrial EGFR pathway and apoptosis. These results also suggest that tumor cells may re-program their intracellular trafficking of EGFR by increasing its mitochondrial accumulation, as a mechanism for escape from therapy- and stress-induced apoptosis and growth suppression. The exact mechanisms by which mitochondrial EGFR modulates apoptotic response are currently being investigated in our laboratory.

## Methods

### Cell lines and cell culture

Human GBM T98G and U87MG cells, human breast carcinoma MDA-MB-468 cells and Chinese hamster ovary (CHO) cells were obtained from American Type Culture Collection (Manassas, VA). U87MG-EGFRvIII stable transfectant cells were established from the parental U87MG cells that expressed a very low level of wild-type EGFR as previously described [19]. All cell lines were cultured in DMEM with 10% fetal calf serum while the U87MG stable transfectant cell lines were additionally supplemented with 0.7 mg/ml G418.

### Reagents and chemicals

All chemicals were purchased from Sigma (St. Louis, MO) unless otherwise stated. Rabbit polyclonal antibody against EGFR (sc-05) used in western blotting was purchased from Santa Cruz Biotech. (Santa Cruz, CA). Monoclonal mouse EGFR antibody used in immunofluorescence staining was obtained from Zymed. Mouse monoclonal Myc-tag antibody was from Roche (Indianapolis, IN). Lamin B mouse monoclonal antibody was from Calbiochem (San Diego, CA). Mouse monoclonal β-actin and α-tubulin antibodies were obtained from Sigma. Rabbit polyclonal Cox IV antibody was from Abcam (Cambridge, MA). Mouse monoclonal anti-EGFR antibody used in immunofluorescence staining was from Zymed/Invitrogen (Carlsbad, CA). Mitotracker was purchased from Invitrogen/Molecular Probes. All transfections were performed with cells in exponential growth using lipofectamine 2000 (Invitrogen) and FuGENE HD (Roche). Iressa was purchased from LC Laboratories (Woburn, MA). CellTiter Blue Cell Viability Assay was obtained from Promega (Madison, WI). Mitochondrial isolation kit was from Pierce (Rockford, IL). QuikChange Mutagenesis Kit was from Stratagene (Madison, WI).

### Determination of EGFR/EGFRvIII mitochondrial translocalization

For this, mitochondria were isolated from tumor cells using a commercial kit (Pierce), according to the manufacturer's instructions. Both mitochondrial and non-mitochondrial fractions were subjected to protein extraction using 1% SDS and 0.1% NP-40 and sonication followed by centrifugation at 15,000 × g and 4°C for 20 min. In these studies, we subjected 25% of the mitochondrial proteins and 2.5% of the non-mitochondrial proteins to western blotting. Band signals from mitochondrial EGFR/EGFRvIII and non-mitochondrial EGFR/EGFRvIII were determined densitometrically using the NIH ImageJ software, as we previously described [49], and normalized against loading controls, Cox IV and β-actin/α-tubulin. The extent of EGFR/EGFRvIII mitochondrial translocalization, designated as mtEGFR/EGFRvIII Index, was computed using the equation: (mitochondrial EGFR/EGFRvIII ÷ 25%)/(mitochondrial EGFR/EGFRvIII ÷ 25%) + (non-mitochondrial EGFR/EGFRvIII ÷ 2.5%).

### Detection of mitochondrial EGFR/EGFRvIII via immunofluorescence staining and confocal microscopy

Tumor cells seeded in 8-well Lab-Tek chamber slides (Nunc Inc., Rochester, NY) for 24 hrs were washed with ice-cold PBS, fixed in 4% paraformaldehyde for 15 min and permeablized with 0.2% Triton-X100 for 5 min. Following treatment with 10% normal goat serum/1% BSA for 60 min, the cells were incubated with polyclonal rabbit Myc or monoclonal mouse EGFR antibody overnight at 4°C. After three washes with PBS, the cells were incubated with goat anti-rabbit secondary or donkey anti-mouse antibodies (1:200, Vector Lab) tagged with Texas Red or fluorescein, respectively. To label the mitochondria, the cells were additionally exposed to DMEM containing 200 nM mitotracker for 30 min prior to fixation in 4% paraformaldehyde. They were then mounted with VECTASHIELD Mounting Medium with and without propidium iodide (for nuclei detection) and examined under a Zeiss LSM 510 confocal microscope.

### Detection of mitochondrial EGFR by transmission electron microscopy (EM)

MDA-MB-468 breast cancer cells were subjected to EM analysis. Briefly, following incubation with mouse IgG, cell sections were treated with mouse monoclonal EGFR antibody. The cells were then incubated with gold particle (15 nm) labeled goat anti-mouse secondary antibody (Amersham Biosciences, USA) for 45 min. Sections were washed, stained with uranyl acetate for 2 min and Reynolds's lead citrate for 1 min and examined in a Jeol 1200EX microscope.

### Generation of mutant EGFR and EGFRvIII with enriched mitochondrial presence using site-directed mutagenesis

This was performed using QuikChange Mutagenesis Kit (Stratagene) and PCR, according to the manufacturer's instructions [24]. The plasmids carrying EGFR and EGFRvIII cDNAs, namely, pCMV-Tag5A-EGFR and pCMV-Tag5A-EGFRvIII [12], were used as templates in the PCR-mutagenesis reactions. Primers used to generate EGFR-MTS1 and EGFRvIII-MTS1 mutants are 5'-ATGCCTTTGAGAACGCAGAAATCGCACGCGGCAGGA-3' (forward) and 5'-TCCTGCCGCGTGCG ATTTCTGCGTTCTCAAAGGCAT-3' (reverse). To produce EGFR-MTS2 and EGFRvIII-MTS2, primers with the sequences of 5'-GATTACGCTCCGCAAGGAGGCAAGTGATGGAGATG-3' (forward) and 5'-CATCTCCATCACTTGCCTCCTTGGCGGAGCGTAATC-3' (reverse) were used. Subsequently, the EGFRvIII-MTS1 and EGFRvIII-MTS2 constructs were transfected into U87MG GBM cells to establish U87MG-EGFRvIII-MTS1 and U87MG-EGFRvIII-MTS2 stable transfectant cell lines.

### Nuclear fractionation and western blotting

This was performed as previously described [24,50]. Briefly, serum-starved cells treated with EGF (100 ng/ml) for 0 and 10 mins were collected, washed with PBS, and swelled in hypotonic buffer (25 mM Tris-HCl, pH 7.5, 5 mM KCl, 0.5 mM dithiothreitol, 1 mM PMSF and 0.15 m/ml aprotinin) for 20 min on ice. Following homogenization using a Dounce homogenizer, nuclei were pelleted and washed. Ultrasonic disruption was used to extract nuclear proteins from the isolated nuclei. To isolate non-nuclear extracts, the supernatant was exposed to 1% SDS and 0.1% NP-40, centrifuged at 15,000 g to remove cell debris and the resulting supernatant collected. Western blotting was conducted. Histone H3 serves a nuclear marker.

### Determination of half-life of EGFRvIII and its MTS mutants

Tumor cells exposed to 10 ug/ml cycloheximide for 0, 30, 60, 120 and 180 min were harvested, total proteins extracted and subjected to western blotting. A Myc-tagged mouse monoclonal antibody (Roche) was used to detect Myc-tagged EGFRvIII and EGFRvIII-MTS fusion proteins. Membranes were also blotted with an anti-α-tubulin antibody (Sigma).

### TUNEL assay for apoptosis

This was performed using a TUNEL assay kit (Invitrogen), according to the manufacturer's instructions [20]. Briefly, the cells were fixed in 70% ethanol, washed and incubated with the labeling solution containing 5-bromodeoxyuridine 5'-triphosphate and deoxynucleotidyl terminal transferase for 1 hr at 37°C. The DNA incorporated bromodeoxyuridine was detected by Alexa Fluor^® ^488 dye-labeled anti-BrdU antibody at room temperature for 30 min. The cells were then counterstained with propidium iodide and observed under a Zeiss LSM 510 confocal microscope. Yellow signals indicate nuclear fragmented DNA, the merged products of fragmented DNA (green fluorescence) and nuclei (red fluorescence). A total of 250-300 cells were examined in each experiment and three independent experiments were conducted to derive means and standard deviations. Extent of apoptosis was subsequently computed using the equation, (# of nuclei with fragmented DNA)/(# of total nuclei).

### Cell survival and clonogenic growth assays

Tumor cells in exponential growth were seeded in 96-well culture plates and treated with vehicle control (1% DMSO) and various agents. After 48 hrs, the cells were subjected to cell survival analyses using the CellTiter Blue Cell Viability Assay (Promega). Briefly, 25 μl of the CellTiter Blue reagent was added to each well containing 100 ul media, incubated for 4 hrs at 37°C, and then the absorbance measured at 560 nm/590 nm using a plate reader (Synergy-HT, BIO-TEK, Winooski, VT). Clonogenic growth assay was performed in 6-well cell culture plates with 1,000 cells seeded per well as previously described [19]. Seeded cells were treated with 1% DMSO or 12.5 μM Iressa in 1% DMSO for 24 hrs, medium removed and replaced with fresh drug-free growth medium for 10-14 days. Colonies were stained with crystal violet blue solution (Sigma) for 1 hr, washed with water, dried and counted. Triplicate wells were used for each treatment and three independent experiments were performed to derive means and standard deviations.

### Statistical analysis

Student *t*-test was performed using STATISTICA (StatSoft Inc., Tulsa, OK) and Microsoft Excel.

## Competing interests

The authors declare that they have no competing interests.

## Authors' contributions

XC carried out mitochondrial and nuclear fractionations, western blotting studies and determination of protein half-life and participated in the cancer cell culture. HZ performed mutagenesis and generation of stable transfectant cells, and carried out the immunofluorescence staining and confocal microscopy and cell survival assays. FAO helped to revise the manuscript. HWL conceived of the study, designed and coordinated the project and wrote the manuscript. All authors read and approved the final manuscript.
